# The prevalence and causes of pediatric uncorrected refractive error: Pooled data from population studies for Global Burden of Disease (GBD) sub-regions

**DOI:** 10.1371/journal.pone.0268800

**Published:** 2022-07-01

**Authors:** He Cao, Xiang Cao, Zhi Cao, Lu Zhang, Yue Han, Changchun Guo

**Affiliations:** 1 Department of Ophthalmology, Shenzhen People’s Hospital (The Second Clinical Medical College, Jinan University, The First Affiliated Hospital, Southern University of Science and Technology), Shenzhen, Guangdong Province, People’s Republic of China; 2 Emergency Department, The First Affiliated Hospital of Jiamusi University, Heilongjiang Province, People’s Republic of China; 3 VPL Department, Mentor Graphics Technology (Shenzhen) CO. LTD., Guangdong Province, People’s Republic of China; 4 Department of Ophthalmology, No. 4 Hospital of Xi’an City, Xi’an, Shanxi Province, People’s Republic of China; 5 Nangang Branch, The Second Hospital of Heilong jiang Province, Harbin City, Heilongjiang Province, People’s Republic of China; 6 Pingshan District People’s Hospital of Shenzhen, Shenzhen, Guangdong Province, People’s Republic of China; L V Prasad Eye Institute, INDIA

## Abstract

**Background:**

There are limited systematic reviews on the prevalence of uncorrected refractive errors in children. We aimed to summarize the prevalence and causes of pediatric uncorrected refractive error (URE) from studies in the Global Burden of Disease (GBD) sub-regions.

**Methods:**

The pooled analysis used the individual participant data (ages less than 20 years old) from population-based studies around the world by regions. URE was defined as presenting VA < 6/18 and improving to ≥ 6/18 or ≥1 line on using a pinhole in either eye, with main causes of myopia, hyperopia or astigmatism. Each study provided data on any URE, myopia, hyperopia or astigmatism by age, gender, and ethnicity. Prevalence rates were directly age and gender standardized to the 2020 world population with all age groups. Estimates were calculated by study and sub-regions after pooling. Summary estimates included studies in which URE was assessed from a pinhole-corrected refraction in the better eye.

**Results:**

The combined pooled data contained 302,513,219 patients including 8 963 URE cases individuals from 57 studies. Prevalence varied by age and GBD sub-regions and differed by gender. The age- and region-standardized prevalence of URE was 3.41 per 1000 (CI, 1.53~7.62) in Western Pacific region (12 studies), 2.26 per 1000 (CI, 0.85~6.01) in South-East Asia region (14 studies), 5.85 per 1000 (CI, 3.75~9.13) in Americans (11 studies) and 4.40 per 1000 (CI, 3.0~6.45) in Eastern Mediterranean region (13 studies). On the basis of these data, myopia was the first-leading cause in female children with 12~17 age group, with the prevalence rate 18.2 per 1000 (CI, 11.52~23.61). Astigmatism was detected in 27.2 per 1000 male children with 6~11 age group (CI: 19.12–30.68).

**Conclusions:**

Prevalence of URE available data within these sub-regions are widely disparate. Myopia and astigmatism in young age children continue as the leading cause of URE worldwide. Providing appropriate refractive correction to those individuals whose vision can be improved is an important public health endeavor with implications for safety and quality of life.

## Background

The World Health Organization’s Global Action Plan for 2014 to 2019 has identified human resources for refractive error as a priority to reduce avoidable blindness globally [1]. It highlights the need for regional surveys to generate evidence on the magnitude and causes of visual impairment (VI) [2]. Uncorrected refractive error (URE) is the most common cause of VI in children [3–6]. According to the World Health Organization (WHO), approximately 19 million children and adolescents 5 to 15 years of age suffer from VI, among which, approximately 12.8 million cases (67%) are due to URE [5]. Uncorrected refractive error may reduce educational opportunities, productivity, and overall quality of life [7].

Identification of the prevalence and causes of visual impairment are crucial for the establishment of local programs and supra-national, continental and world prevention strategies. This information is of critical importance for both scientists and international agencies working in the field. Over the past decade, population-based surveys conducted in different regions and all age groups had revealed great disparity in prevalence of URE. These factors might include genetics, environmental factors, and socio-economic status [8, 9]. There has been many population-based studies from different regions in the last two decades on various eye conditions, and there are many reports published with the aim of determining the prevalence of URE among various age groups in the whole world [10–12]. However, the reported prevalence varies considerably between studies due to differences in the study populations, methodologies, and definitions of conditions studied [13]. Population-based pooled estimates provide evidence for policy decisions. Herein, there are limited systematic review on the prevalence of uncorrected refractive errors in children, especially for meta-analysis. The present study sought to evaluate the epidemiologic patterns of uncorrected refractive error in children, using available data from all population-based study. Findings from this study might be useful in developing studies to prevent pediatric visual impairment from URE.

## Methods

### Search strategy

A systematic review of the literature for all relevant population-based studies that undertook uncorrected refractive error was conducted. We searched all English language and human subject articles using a keyword search of by MEDLINE (1950 to September 30, 2021), EMBASE (1966 to September 30, 2021), Web of Science (1900 to September 30, 2021), Cochrane library (including the Cochrane Central Register of Controlled Trials, 1800 to September 30, 2021), and abstracts from the Association for Research in Vision and Ophthalmology (January 1962 to September 30, 2021), with the following search terms: [(uncorrected refractive error OR under-corrected refractive error OR correctable visual impairment OR unmet refractive error AND (prevalence)]. The search strategy used both keywords and Medical Subject Headings (MeSH) terms. A total of 1638 citations were identified as of December 31, 2020. This study was approved by the ethics committee of Southern University of Science and Technology and conformed to requirements of the tenets of the Declaration of Helsinki.

Retrieved studies were imported into Refworks (version 1.0; Refworks, Bethesda, MD). Duplicate articles appeared twice or more, whether in the same or different databases were deleted. Data extraction and evaluation of study quality were performed independently by two reviewers; any disagreements were resolved by discussion with the senior investigators. The bibliographies of the full text articles that were reviewed were searched for relevant references. Full-text articles were then obtained based on the initial screening of abstracts and the data extraction form was completed. The full texts of the remaining studies were then read to determine whether they met our inclusion criteria. In addition, the reference lists from all identified studies were examined.

### Inclusion and exclusion criteria

**Fig 1:**
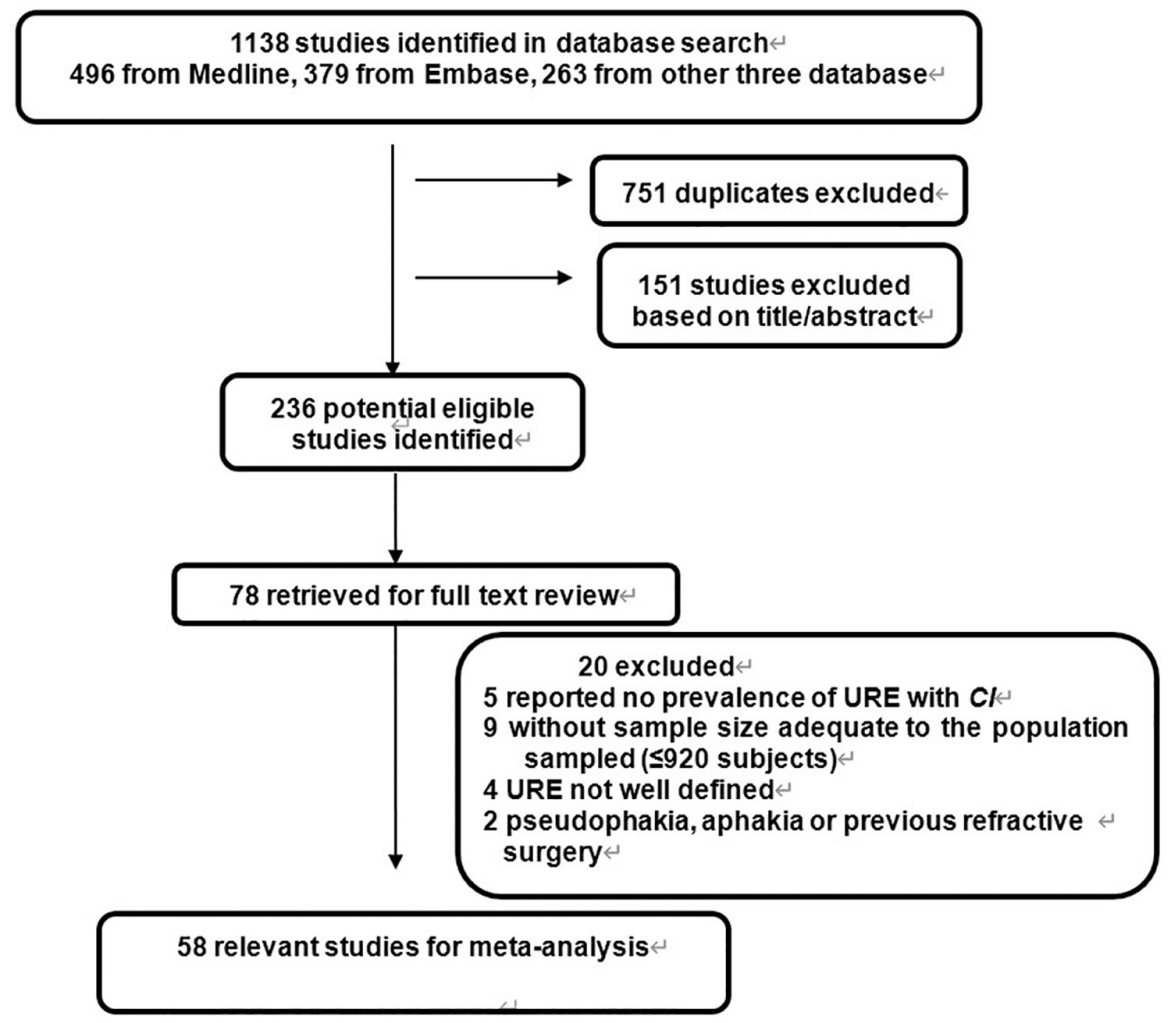
Flow diagram showing the selection process for inclusion of studies in the meta-analysis. *CI* = confidence interval; URE = uncorrected refractive error.

The full text of the remaining articles was reviewed to ensure all studies met the inclusion criteria and did not meet the exclusion criteria. Studies were included if they (i) have to be population based, representative of the country and of the area sampled, with sample size adequate to the population sampled (from 1100 to 1.4 million); (ii) sufficient response rate (80% or higher); (iii) reported a prevalence of with 95% confidence interval (CI), or allowed for the calculation of it from the raw data presented in the article, and (iv) reported data for persons, with definitions of URE and visual impairment in agreement with those used in this study. (v) reported presenting visual acuity (PVA) <6/18 with its causes and provided the standard World Health Organization categories of visual acuity (VA); (vi) In children, refractive diagnostics had to be determined by objective refraction under cycloplegia plus subjective refraction. We excluded (i) they were not population-based or without sample size adequate to the population sampled (i.e., excluding studies of clinic patient samples), (ii) the prevalence of URE with 95% CI was not reported or could not be calculated, (iii) URE or visual impairment were not clearly defined. (iv) Participants with bilateral pseudophakia or aphakia and those who had undergone refractive surgery were also excluded. In total, 58 population-based studies had prior institutional review board approval and provided appropriately de-identified data for analysis (Fig 1).

### Definition of uncorrected refractive error

All included studies had URE by a standardized protocol.

Definition of URE: PVA was measured using a LogMar chart at four meters from the subject who wore their usual distance correction. Among those with a PVA <0.3 (equivalent Snellen PVA<6/12), those who improved ≥1 line with a pinhole-corrected refraction in their better eye were classified as having URE. The definitions of visual impairment used for the estimates in this study follow the categories of the International Classification of Diseases Update and Revision 2006 that defines impairment according to presenting vision.

### Data extraction and appraisal of quality of studies

For each study, the following characteristics were extracted: (i) study region, (ii) year of publication, (iii) sample size, (iv) male ratio, (v) age group, (vi) ethnicity of subjects in the analysis, (vii) definition of URE, (viii), (ix) prevalence of URE with CI, (x) quality criteria.

The quality of all studies was assessed for the following attributes according to the quality assessment criteria reported by de Weerd et al [13] and Sophie Rogers et al [14]: (I) Participants selected should ideally by representative of the general population. Methods of achieving this may involve using population registries, inhabitants of a defined area, and people registered with a general practice. Participants attending health checkups may be biased and only cover certain population groups. (ii) Appropriate recruitment of the population. Recruitment was considered appropriate if recruitment of participants was random or consecutive rather than performed for convenience. (iii) Adequate response rate (>80%). (iv) Objective documentation of the outcome, in this case, documentation by retinal photography performed according to standardized protocols and graded according to standard definitions.

A score of 3 or higher was considered adequate quality (Table 1).

**Table 1:** Characteristics of included population-based studies.

Study name ^ref^	GBD sub-region	Publish year	N subjects (participation rate %)	Male (%)	Age group (years)	Ethnicity ^b^	Definition of URE	Prevalence % (95% CI) of URE	No. of Quality Criteria ^d^
							Definition 1^c^		
							Definition 2^c^		
							Definition 3^c^		
Israeli ^60^	EURO A	2008	1862(88.1)	52.7	6~14	Arabs 66.3% Jews 33.7%	Def1	2.6 (1.8~3.9)	3
Southern California ^28^	EURO B2	2011	11332(89.2)	53.5	6~12	Latino61.5%, Asian13.3% African American12.5%	Def1	7.6 (5.2~9.1)	3
Tanzania ^11^	AFRO E	2015	502(99.7)	57.2	15~19	Tanzania	Def1	3.64 (1.35~9.86)	3
South Africa ^78^	AFRO E	2015	400(92.1)	40.3	6–18	South Africa	Def1	10.64 (8.62~12.37)	3
Iran ^22^	EMRO B	2011	11975 (90.4)	45.1	8.9~46.7	Iran	Def1	1.16 (0.86~1.36)	4
Iran ^31^	EMRO B	2017	747(86.5)	43.5	0~20	Iran	Def1	0.75 (0.12~1.38)	3
Iran ^38^	EMRO B	2010	2130(87.9)	51.9	7~15	Iran	Def1	5.56 (4.66~6.22)	4
Iran^40^	EMRO B	2015	3475(45.8)	47.7	1~20	North of Iran	Def1	0.98 (0.49~1.47)	3
Iran ^42^	EMRO B	2009	14000000(67.0)	50.3	3~6	Iran	Def1	3.82 (3.79~3.85)	5
Iran ^59^	EMRO B	2016	4614(89.0)	52.2	7	Iran	Def1	8.89 (7.65~10.13)	3
Iran ^62^	EMRO B	2017	4614(89.1)	51.8	7	Iran	Def2	8.49 (7.65~9.39)	
Iran ^70^	EMRO B	2007	5544(96.9)	44.0	7~18	Iran	Def1	1.7 (1.10~2.23)	4
Iran ^74^	EMRO B	2006	4353(70.3)	41.6	≥5	Iran	Def3	4.8 (4.10–5.40)	4
Iran ^76^	EMRO B	2004	4565 (70.3)	41.8	1~99	Asia	Def1	0.85 (0.70–1.00)	5
**Tunisia** ^ **49** ^	EMRO B	2016	6192(88.1)	46.7	6~14	Tunisia	Def1	2.61 (2.47~2.77)	4
Tunisian ^68^	EMRO B	2015	6192(88.1)	51.7	6`14	Tunisia	Def1	6.67 (5.68~7.69)	4
Saudi Arabia ^88^	EMRO B	2014	5176(90.3)	49.7	6·13	Arabia	Def2	16.3 (15.3~17.3)	3
Egypt ^43^	EMRO D	2015	2070(92.5)	56.0	7~13	South Sinai and Bedouin	Def1	29.4 (21.5~35.2)	5
Thailand ^33^	SEARO B	2010	2340(88.6)	48.3	6~12	Thailand	Def1	9.0 (8.2~9.4)	4
Singaporean ^37^	SEARO B	2010	2017(72.3)	50.9	1.25~6	Singaporean Chinese	Def3	1.44 (0.94~1.89)	4
**Malaysia** ^**51**^	SEARO B	2008	705(83.9)	51.9	6~12	Malaysia	Def1	7.0 (5.66~8.22)	3
Malaysia ^80^	SEARO B	2002	18027 (95.1)	47.0	0.08–96	Malay (54.0)) Chinese (24.0) Indian (6) Indigenous (10)	Def1	1.19 (1.09–1.29)	5
Sri Lanka ^54^	SEARO B	2009	14669(73.0)	52.1	6~15	Sri Lanka	Def1	27.0 (23.79~30.85)	3
Indonesia ^79^	SEARO B	2003	989 (83.4)	46.6	21–88	Asia	Def1	0.75 (0.54–0.95)	3
South India ^27^	SEARO D	2011	3095 (94.0)	52.5	15~49	India	Def3	3.6 (3.0~4.1)	4
India ^39^	SEARO D	2017	4329 (95.0)	48.5	6~20	India	Def2	3.29 (2.15~4.61)	4
India ^41^	SEARO D	2009	3300(97.0)	53.3	15~50	India	Def3	2.7 (2.1~3.2)	4
India ^48^	SEARO D	2009	3214(97.0)	47.5	7~15	India	Def1	2.1 (1.8~2.4)	3
India _67_	SEARO D	2016	2178(92.0)	51.0	0~15	India	Def1	1.2 (1.1~2.4)	3
India ^81^	SEARO D	2002	6447 (92.0)	51.9	5–15	Asia	Def3	5.59 (3.74–7.38)	4
India ^82^	SEARO D	2002	4074 (92.3)	51.9	7–15	Asia	Def3	1.96 (1.37–2.44)	4
Nepal ^64^	SEARO D	2008	4501(95.1)	49.2	11~15	Nepal	Def1	0.80 0.69~0.92)	3
Nepal ^85^	SEARO D	2019	76588(95.0)	48.7	0~15	Nepal	Def1	0.12 (0.11~0.13)	4
Bangladesh ^87^	SERRO D	2019	33549(93.1)	50.5	0~15	Nepal	Def1	3.24 (3.11~3.45)	4
Australia ^19^	WPRO A	2012	2899(87.0)	50.0	6–11	Australia	Def1	6.57 (5.91~7.22)	4
Australia ^36^	WPRO A	2010	920(69.1)	45.6	16~89	Australia	Def1	21.1 (14.4~27.6)	4
China ^9^	WPRO B1	2015	1255(89.9)	54.5	3~6	China	Def1	1.7 (1.5~1.9)	4
China ^77^	WPRO B1	2004	4364 (86.4)	51.9	5–15	Han ethnicity	Def1^G^	1.28 (0.99–1.67)	4
China ^10^	WPRO B1	2014	9673(98.3)	57.9	7–12	China	Def1	0.58 (0.28~1.28)	4
China ^18^	WPRO B1	2015	6321(79.5)	50.8	6~16	China	Def1	29.69 (25.32~32.10)	5
China ^21^	WPRO B1	2016	8398(98.4)	54.0	3~10	China	Def1	19.8 (14.8~24.7)	5
China ^32^	WPRO B1	2015	690(94.0)	51.6	4~19	Uyghur, Han, Hui ethnicity	Def1	19.3(13.0~27.0) myopia 7.0% (4.0~12.0) astigmatism	5
China ^83^	WPRO B1	2015	5862(94.8)	53.3	3~6	China	Def1	0.51 (0.44~0.68)	5
Vietnam ^26^	WPRO B2	2012	28800(97.4)	52.2	0~15	Vietnam	Def1	0.3 (0.2~0.4)	5
Cambodia^19^	WPRO B2	2012	6156(89.8)	45.4	12~14	Cambodia	Def1	3.3 (1.32~4.15)	
Cambodia ^66^	WPRO B2	2007	5803(88.5)	43.9	all age groups	Cambodia	Def1	2.2 (1.9~2.5)	5
**Fiji** ^**30**^	WPRO B3	2011	8201(91.0)	46.5	12~20	Fiji	Def3	0.9 (0.7~1.1)	3
**USA** ^**16**^	AMRO A	2017	1538(93.0)	52.5	3~6	Overall race/ethnicity	Def1	1.04 (0.83~1.24)	3
**USA** ^**46**^	AMRO A	2009	3207(77.0)	48.9	1.25~6	Hispanic 50.0%, African-American50.0%	Def2	0.9 (0.1~1.7)	4
USA ^71^	AMRO A	2006	13265(93.4)	51.6	≥12	USA	Def1	5.3 (5.0~5.7)	5
USA ^72^	AMRO A	2015	7753(95.6)	47.4	3~20	USA	Def1	3.5 (3.2~5.3)	4
Brazil ^44^	AMRO B	2009	6119(92.1)	43.2	all age groups	Brazil	Def1	4.4 (3.6~5.5)	5
Brazil ^45^ The Botucatu Study	AMRO B	2009	2485(75.3)	42.5	1~91	European 80.6% Other races 14.1% African-Brazilian 4.9%	Def1	3.8 (3.1~4.4)	4
Brazil ^55^	AMRO B	2008	2441(86.4)	46.6	11~14	Brazil	Def1	7.5 (5.9~9.0)	3
Six countries ^63^	AMRO B	2008	40779(87.9)	49.3–51.9	5~15	Asia, Africa, Latin America	Def1	15.2 (11.6~19.5)	5
Mexico ^17^	AMRO B	2017	136312(88.2)	50.0	6~19	Mexican	Def1	57.8 (32.4~65.8)	3
Mexico ^60^	AMRO B	2008	2533(89.1)	47.2	6~20	Mexican	Def1	10.5 (8.2~12.5)	3
Paraguay ^50^	AMRO B	2017	1466(90.3)	49.9	3~22	Latin America	Def1	6.11 (5.3~8.1)	1

(EPIC) a: The European Prospective Investigation of Cancer. Ethnicity b Totals for ethnicity may not equal 100%; some studies had subjects from other ethnic groups not included in this analysis

Definition 1 & 2 & 3 were all included by the Definition of URE.

Definition1 c: Presenting visual acuity (PVA) was measured using a LogMar chart at four meters from the subject who wore their usual distance correction. Among those with a PVA <0.3 (equivalent Snellen PVA<6/12), those who improved ≥1 line with a pinhole-corrected refraction in their better eye were classified as having URE. Refractive error was measured using an autorefractor without cycloplegia. A spherical equivalent of -0.5 diopter (D) or worse was defined as myopia, +2.0 D or more was defined as hyperopia, and a cylinder refraction greater than 0.75 D was considered astigmatism.

Definition2 c: Presenting visual acuity (PVA) was measured using a LogMar chart at four meters from the subject who wore their usual distance correction. Among those with a PVA <0.3 (equivalent Snellen PVA<6/12), those who improved ≥2 line with a pinhole-corrected refraction in their better eye were classified as having URE.

Definition3c: Decreased VA was defined as visual acuity worse than 20/50 (logMAR 0.4) for children 30~47 months (<4 years) of age and worse than 20/40 (logMAR 0.3) for those 48~72 months (4~6 years) of those who were testable.

The No. of quality criteria d: 1 = sample size adequate to the population sampled, with an appropriate recruitment of the population (random or consecutive), 2 = sufficient response rate (≥80%), 3 = reported a prevalence of with 95% confidence interval (CI), or allowed for the calculation of it from the raw data presented in the article, 4 = objective outcome, reported data for persons, with definitions of URE and visual impairment in agreement with those used in this study, moreover, reported presenting visual acuity (PVA) <6/18 with its causes and provided the standard World Health Organization categories of visual acuity (VA), 5 = in children, refractive diagnostics had to be determined by objective refraction under cycloplegia plus subjective refraction.

### Statistical analysis

Data from each study were checked for consistency in variable definitions before pooling. Sub-regions were categorized as Africa, Asia, Americas, Eastern Mediterranean, Europe, South-east Asia, Western-Pacific (Table 2). Study-specific and pooled-data estimation of URE prevalence rates were obtained using the direct method of age-gender-standardization to the 2020 world population with all age groups younger than 20 years old [15]. This standardization involved 4 age categories (0–5 years, 6–11 years, 12–17 years and 18–20 years) in both genders. The calculation of 95% confidence intervals (CIs) for the standardized prevalence rates used a normal approximation and Breslow-Day standard errors, after being modified to use a binomial assumption for the variance of the crude stratum-specific rates.15 Crude prevalence rates per 1000 and Agresti-Coull modified Wald CIs were also calculated. Initial analyses included data from all 15 studies. Publication bias was evaluated with the use of Egger regression asymmetry test and the Begg’s test. All statistical analyses were performed with Stata version 11.1 (Stata Corp, College Station, TX). A 2-sided P value less than 0.05 was regarded as significant for all analyses.

**Table 2:** Number of included surveys available for Global Burden of Disease (GBD) sub-regions.

GBD sub-regions	WHO member countries	Population b (millions)	Included surveys N
Africa Region			2
AFRO D	Algeria, Angola, Benin, Burkina Faso, Cameroon, Cape Verde, Chad, Comoros, Djibouti, Equatorial Guinea, Gabon, Gambia, Ghana, Guinea, Guinea-Bissau, Liberia, Madagascar, Mali, Mauritania, Mauritius, Niger, Nigeria, Sao Tome and Principe, Senegal, Seychelles, Sierra Leone, Somalia, Sudan, Togo		0
AFRO E	AFRO E Botswana, Burundi, Central African Republic, Congo, Côte d’Ivoire, Democratic Republic of The Congo, Eritrea, Ethiopia, Kenya, Lesotho, Malawi, Mozambique, Namibia, Rwanda, South Africa, Swaziland, Uganda, United Republic of Tanzania, Zambia, Zimbabwe		2
Americans region			11
AMRO A	Anada, United States of America		4
AMRO B	Antigua and Barbuda, Argentina, Bahamas, Barbados, Belize, Brazil, Chile, Colombia, Costa Rica, Cuba, Dominica, Dominican Republic, El Salvador, Grenada, Guyana, Honduras, Jamaica, Mexico, Panama, Paraguay, Saint Kitts and Nevis, Saint Lucia, Saint Vincent and The Grenadines, Suriname, Trinidad and Tobago, Uruguay, Venezuela		7
AMRO D	Bolivia, Ecuador, Guatemala, Haiti, Nicaragua, Peru		0
Eastern Mediterranean region			14
EMRO B	Bahrain, Cyprus, Iran (Islamic Republic of), Jordan, Kuwait, Lebanon, Libyan Arab Jamahiriya, Oman, Qatar, Saudi Arabia, Syrian Arab Republic, Tunisia, United Arab Emirates		13
EMRO D	Egypt, Iraq, Morocco, Yemen		1
Europe region			2
EURO A	Andorra, Austria, Belgium, Croatia, Czech Republic, Denmark, Finland, France, Germany, Greece, Iceland, Ireland, Israel, Italy, Luxembourg, Malta, Monaco, Netherlands, Norway, Portugal, San Marino, Slovenia, Spain, Sweden, Switzerland, United Kingdom		1
EURO B1	Albania, Bosnia and Herzegovina, Bulgaria, Georgia, Poland, Romania, Slovakia, The Former Yugoslav Republic of Macedonia, Turkey, Yugoslavia		0
EURO B2	Armenia, Azerbaijan, Kyrgyzstan, Tajikistan, Turkmenistan, Uzbekistan		1
EURO C	Belarus, Estonia, Hungary, Kazakhstan, Latvia, Lithuania, Republic of Moldova, Russian Federation, Ukraine		0
South-East Asia region			16
SEARO B	Brunei Darussalam, Indonesia, Malaysia, Philippines, Singapore, Sri Lanka, Thailand, Timorese		6
SEARO D	Afghanistan, Bangladesh, Bhutan, India, Maldives, Nepal, Pakistan		10
Western Pacific region			12
WPRO A	Australia, Japan, New Zealand		2
WPRO B1	China, DPR Korea, Mongolia, Republic of Korea		7
WPRO B2	Cambodia, Lao People’s Democratic Republic, Myanmar, Vietnam		3
WPRO B3	Cook Islands, Fiji, Kiribati, Marshall Islands, Micronesia (Federated States of), Nauru, Niue, Palau, Papua New Guinea, Samoa, Solomon Islands, Tonga, Tuvalu, Vanuatu		0
Total			57

GBD sub-regions ^a^ as per the GBD 2000 Project; the letter with each sub-region indicates mortality stratum: A is very low child mortality and low adult mortality, B is low child mortality and low adult mortality, C is low child mortality and high adult mortality, D is high child mortality and high adult mortality, E is high child mortality and very high adult mortality; EURO B and WPRO B sub-divided further to capture epidemiological differences; this classification aims at maximizing the epidemiological homogeneity of sub-regions [7].

Population ^b^ based on United Nations estimates for 2002 [15], as used for the WHO base visual impairment estimates [1].

## Results

In total, data from 58 articles were identified in the final analysis, contributing a sample of some 302,513,219 patients including 8 963 URE cases. Fig 1 shows the phases of article selection.

### Current estimates of gender–standardized prevalence of URE by GBD sub-regions from 2002 to 2020 (Fig 2)

**Fig 2:**
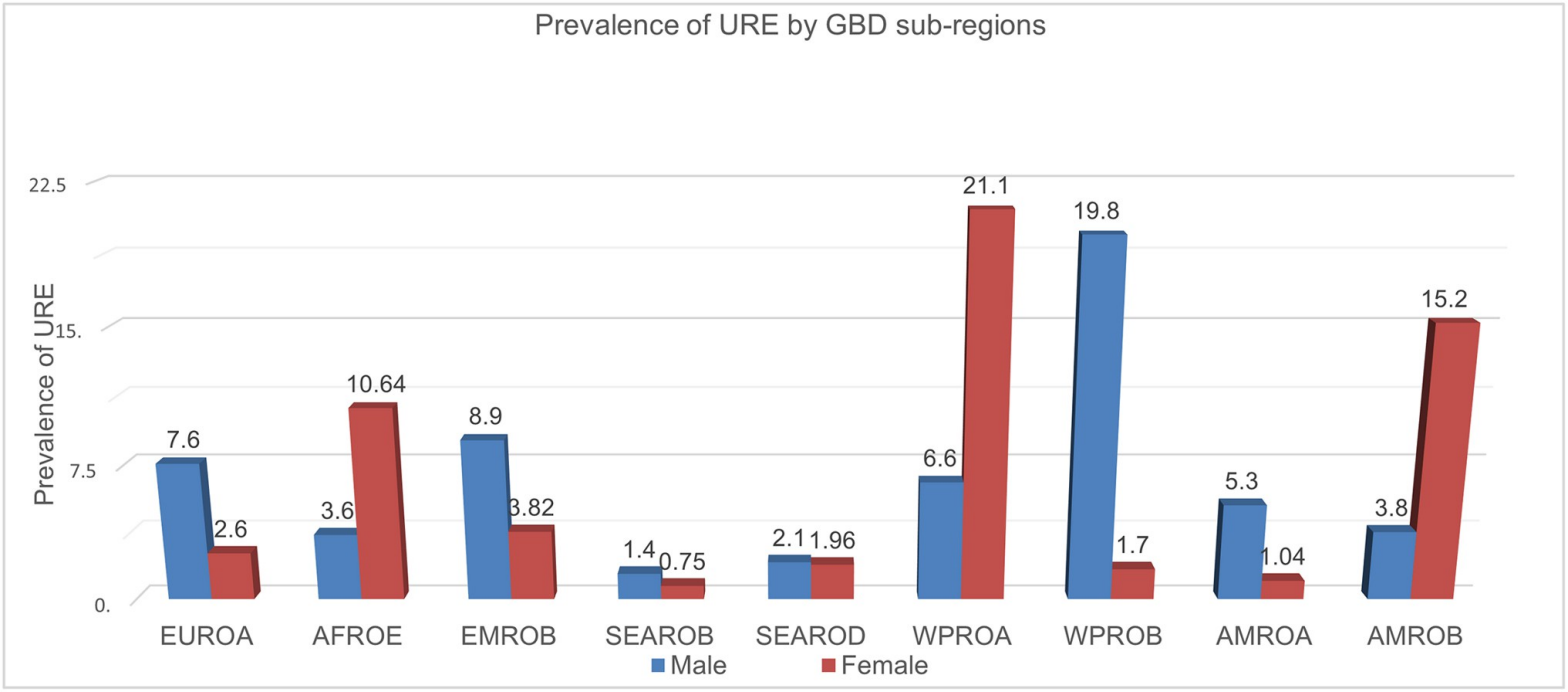
Gender-standardized prevalence of URE by GBD sub-regions. Prevalence rates shown are per 1000 children and include data from only those studies that assessed both eyes for each subject. Prevalence rates have been directly age- and gender-standardized to the 2020 world population with all ages groups Children (< 20 years) (population data extracted from Ref. 15); URE = uncorrected refractive error.

Fig 2 Presents a summary of the results of the surveys available for Global Burden of Disease (GBD) sub-regions. We based these summary estimates on pooled prevalence estimates by age and gender. For children aged younger than 20 years old in WPRO A region, the overall estimated prevalence of URE was 21.1% in female and 6.6% in male respectively. In WPRO B region, Chinese male children presented the major prevalence of URE (19.8 per 1000), which reveal a higher prevalence of URE among Chinese male children than among female children.

In Americas region, Brazil and Mexico female children represented larger prevalence of URE (15.2 per 1000), followed by American and Canadian male children (5.3 per 1000). Most URE cases remained higher prevalence among African female children but to a lower ratio among male children (15.2 vs 3.8).

The south-east Asian children with lower URE cases was projected to shift from 1.4 to 2.1 per 1000 in male children. African female children were projected from 0.75 to 1.96 per 1000.

### Prevalence of URE by GBD sub-regions from 2000 to 2021 (Fig 3)

**Fig 3:**
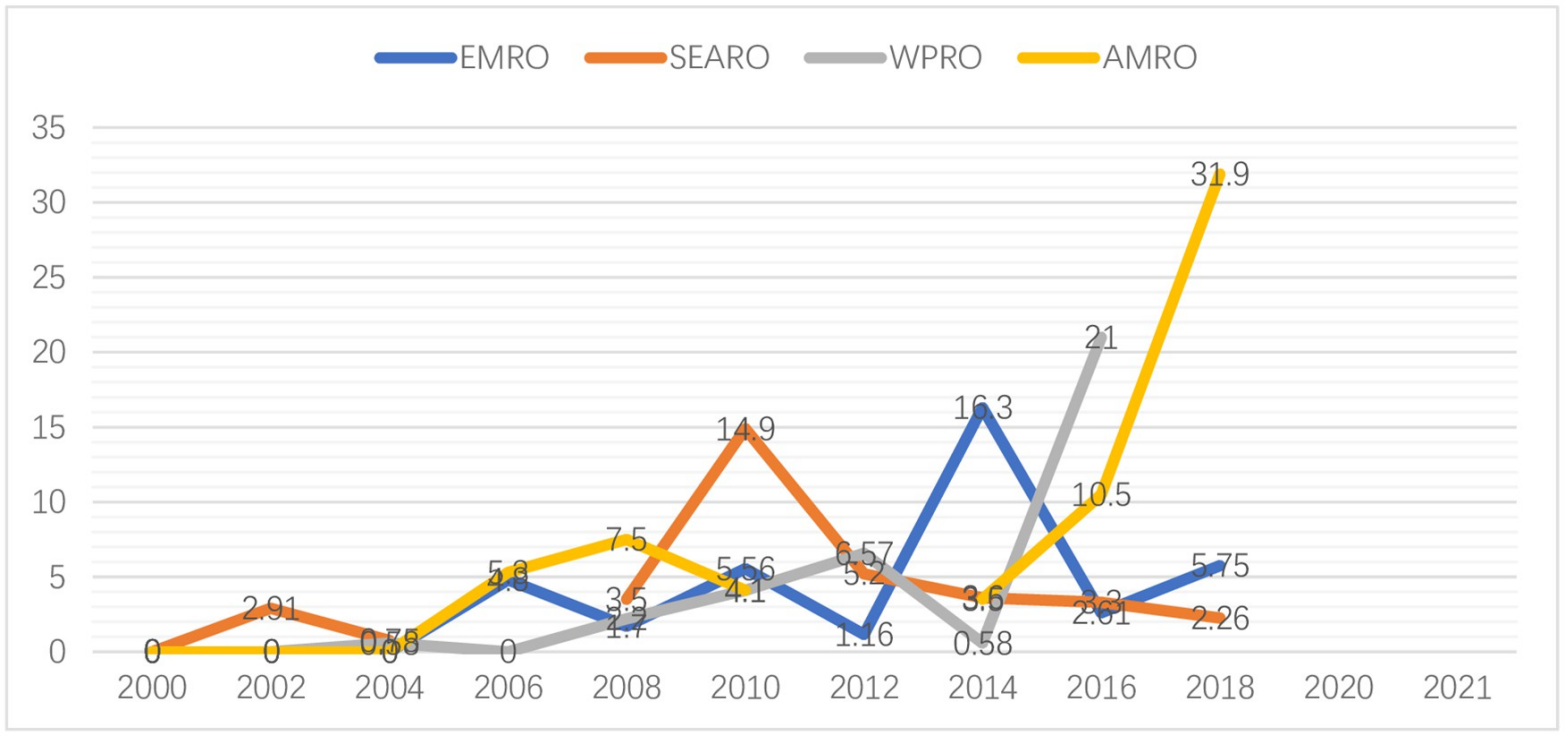
Prevalence of URE by GBD sub-regions from 2000 to 2021 (per/1000).

When summarizing data from the years 2000 to 2021 that allowed for comparisons across GBD sub-regions, it is observed that while the prevalence varies across countries/regions, the overall trend is rising in the whole world.

In Americas region, where the prevalence is already high, the prevalence in children increased sharply from approximately 10.5% to 31.9% between 2016 and 2018. The prevalence in WPRO region increased steadily from 2008 to 2012 from 3.5% to about 6.57% (2001–2010) and then to 21.0% (2016). Although SEARO region has the lowest prevalence, **a transient increasing shift is also observed 2010**.

### Distribution of the estimated number of children with URE in four age groups by gender (Fig 4)

**Fig 4:**
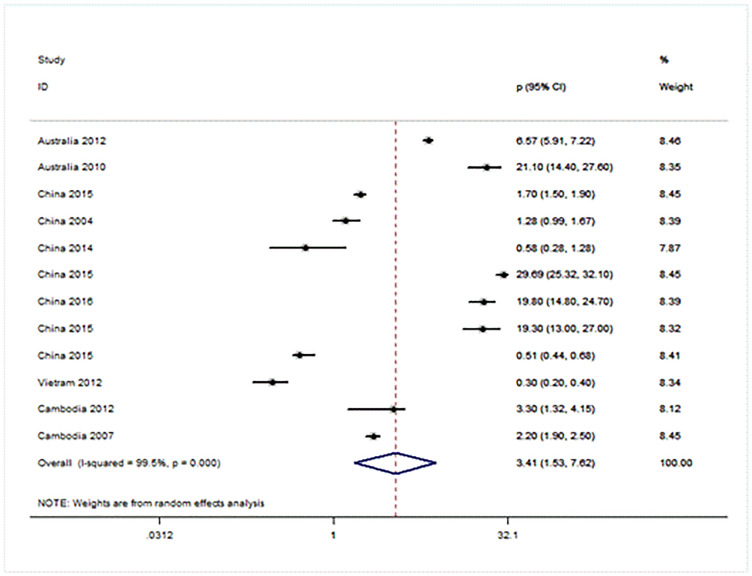
Crude prevalence of myopia, hyperopia and astigmatism by age in gender. The broken line are informative data points from included studies that reported the prevalence of URE.

Fig 3 Shows the prevalence trend of URE in each of the gender of this study. The prevalence of myopia was highest in male children aged 18~20, but there were no significant differences between 6~11 with 12~17 age group (*P* = 0.284). The prevalence of astigmatism and hyperopia was the highest in children aged 6–11. The prevalence of URE were differed significantly with female children; the highest rate of myopia was observed in 12~17 age group and the lowest rate was in 6~11 age group. The prevalence of astigmatism was higher in 6~11 age group (95% CI: 10.43–14.75), continued to 12~17 age group.

### Forest plot showing prevalence of URE among GBD sub-regions (Figs 5–8)

**Fig 5:**
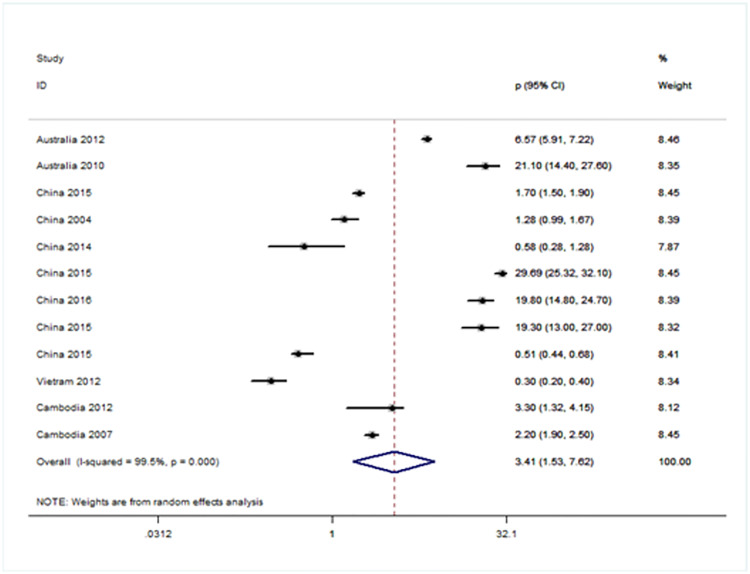
Forest plot showing prevalence of refractive errors among children aged < 20 years in Western Pacific region.

**Fig 6:**
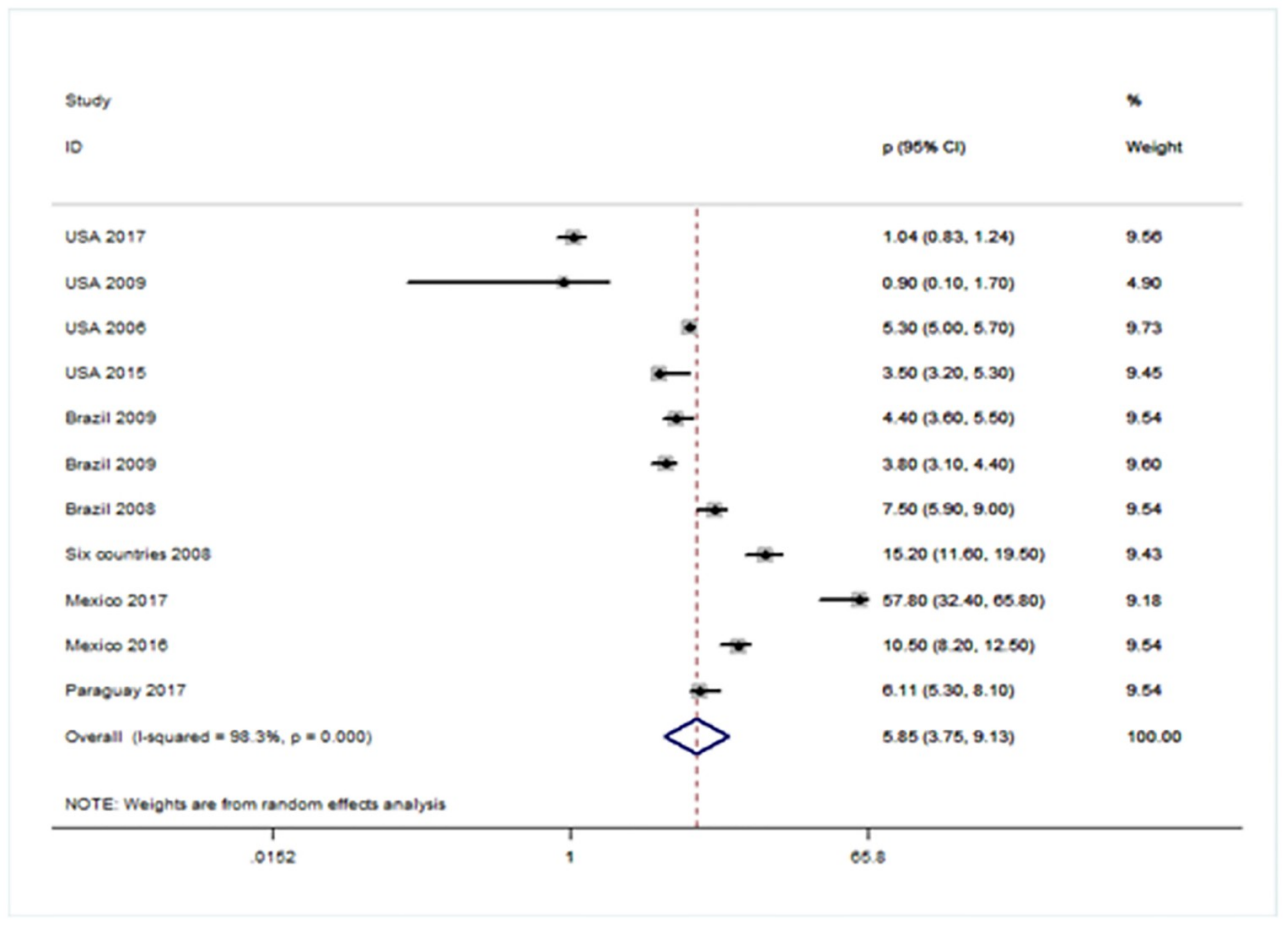
Forest plot showing prevalence of URE among children aged < 20 years in American region.

**Fig 7:**
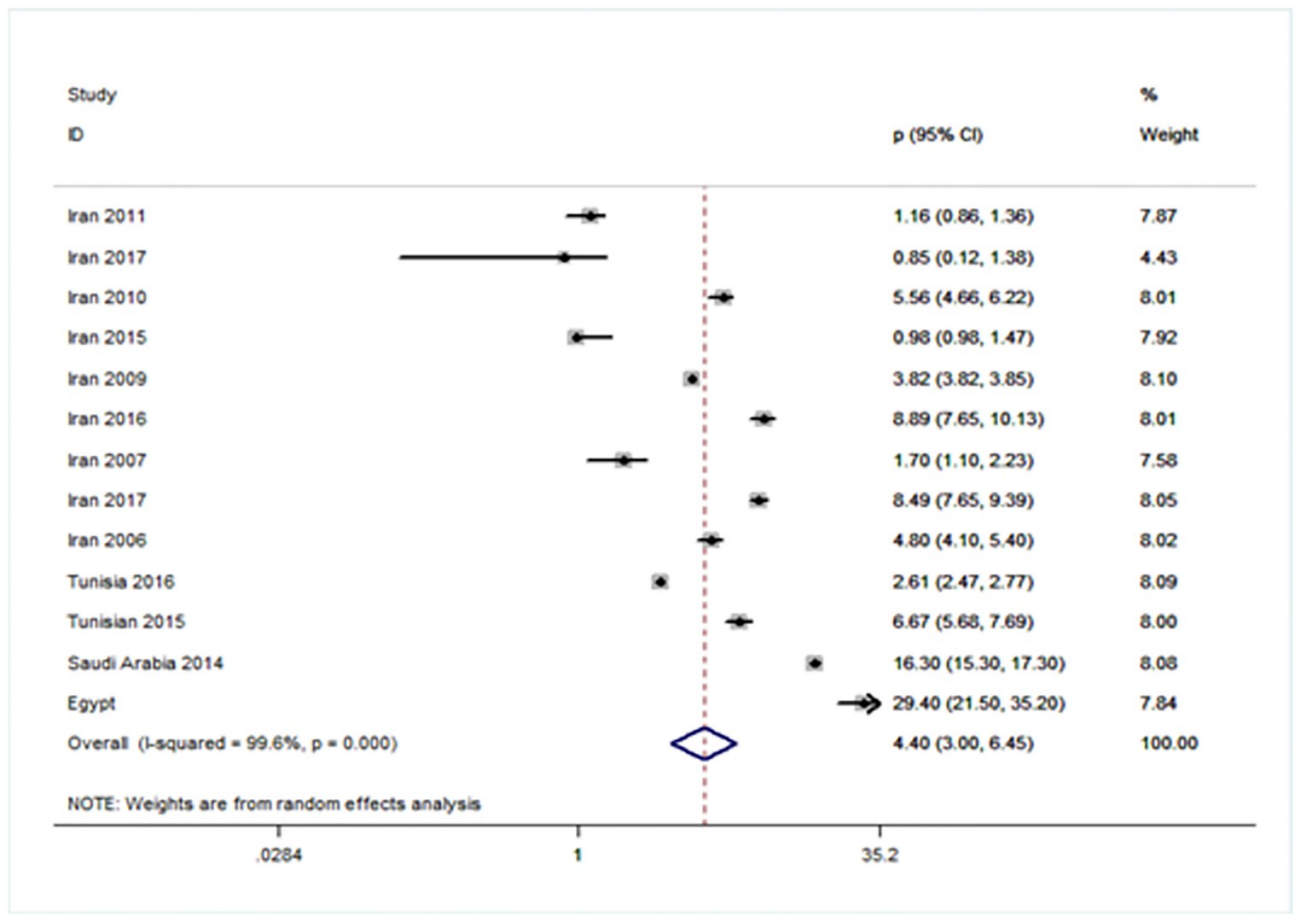
Forest plot showing prevalence of refractive errors among children aged < 20 years in Eastern Mediterranean region.

**Fig 8:**
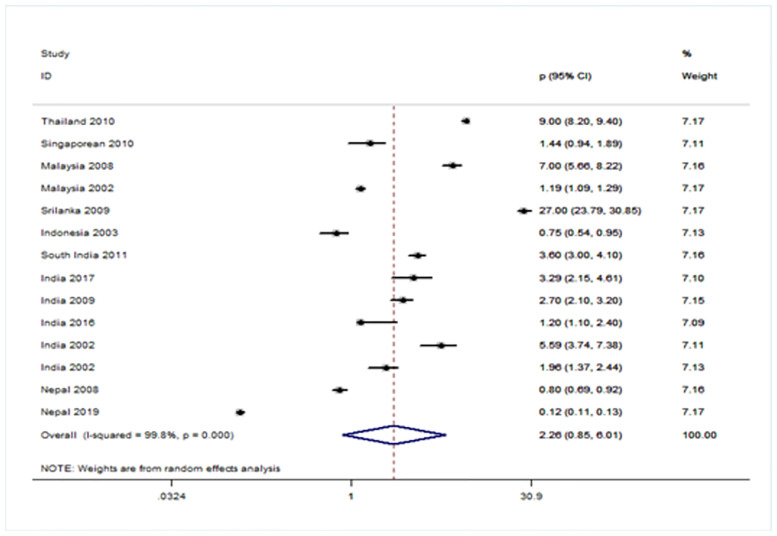
Forest plot showing prevalence of refractive errors among children aged < 20 years in South-East Asia region.

There were four population-based analysis that estimated the prevalence of URE in children. Funnel plots were reviewed for each sub-region and no evidence of publication bias was observed. The findings for each region were summarized in Figs 5 to 8 and discussed separately in the following sections.

Twelve studies were included from WPRO including two from Australia [16, 17], and seven from China [9, 10, 18–23], one each from Vietnam [24] and two studies from Cambodia [16, 25]. The overall prevalence of URE is 3.41 per 1,000 (95% CI: 1.53~7.62). The heterogeneity of the estimates from the included studies was very high (I2 = 99.6%; P = 0.000) [Fig 5].

The prevalence of URE in Americas region is estimated at 5.85 but the confidence limits for this estimate were very wide (95% CI: 3.75~9.13). Four studies were analyzed from the United States of America [26–29] and six studies were analyzed from Brazil [30–32], Mexico [33, 34] and Paraguay [35] [Fig 6].

Estimates of prevalence of URE in EMRO were reported from ten studies from Iran [36–45], one study from Tehran [16], one study from Tehran [46], two studies from Tunisia [47, 48], one study from Saudi Arabia [49], and one study from Egypt [50], contributed to the meta-analysis. The prevalence obtained through analysis of the whole studies was 4.4 (95% CI 3.0∼6.45). The pooled estimate was highly heterogeneous (I^2^ = 99.6%; *P* = 0.000) [Fig 7].

Further, we grouped fourteen studies from SEARO [51–64], and the pooled prevalence was 2.26 (95% CI: 0.85~6.01) [Fig 8]. The heterogeneity of the estimates from the included studies was very high. There was not enough data available to calculate the prevalence from Europe region [65–67], and Africa region [68], which is essential for further planning strategies to address the problem in these two regions.

## Discussion

### Added value of this study

The strengths of this study updates global and regional estimates of causes of URE in children aged less than 20 years until 2020. We examined age-adjusted and gender-adjusted differences in the contribution of these causes to VI. Rapid Assessment of VI studies were disaggregated from prevalence for ages 0~5 years to 17~20 years group, providing more accurate data on age patterns. It may be the only field in pediatric URE which includes reports from almost every region of the world, with an interest in exploring the contribution of causes-eg, myopia, hyperopia and astigmatism-that are known to be of great importance in every population. Furthermore, we did have sufficient data sources to disaggregate URE as a cause of VI.

### Implications of all the available evidence

We noted large differences in the distribution of prevalence and causes of URE by region. Inter-study differences might be related to factors such as the ages of target participants, inclusion criteria, examination methods, publication time and the social-economic forms. The results of different studies in different age groups showed that prevalence of URE ranged from 2.11% in female children in WPROA to 1.08% in male children in WPROB. These two regions had a higher prevalence of URE mostly due to lifestyle, such as increasing near vision task and lack of outdoor activities. This is similar to previous reports [69–72]. Although the prevalence of URE in AMRO had a lower prevalence ranged from 0.38 to 0.53 in male and from 0.10 to 1.52 in female as a cause of race of genetic reasons, it is still relative higher than the prevalence in EMRO and EUROA.

The crude and age-standardised prevalence of URE presented an increasing contribution of myopia in both genders. The results of different studies in different age groups showed that prevalence of myopia ranged from 8.4% in children aged 6 ~11 years to 14.3% in 18~20-year-old male children. The highest prevalence of myopia was about 18.2% in female children and was projected to decline to 14.3% in 18~20 age group. This discrepancy highlights the need for future research into this gender difference and the need to disaggregate between male and female in reporting of research. As mentioned earlier, the lowest prevalence of myopia was seen in South-east Asia [73], and the highest prevalence was seen in the Western Pacific region [21, 67, 74]. With regards to the high incidence rate of myopia in children in WPRO, we believe that the role of environment factors is more important than genetic and ethnic factors. The Refractive Error Study in Children (RESC) reported that the prevalence of myopia was higher in China, compared to Nepal, Chile, India, South Africa, and Malaysia consistent with other reports of high myopia rates among children of east [57, 75–81]. There might be three major factors for myopia formation and development by previous reports: the first is the excessive intensity of near vision work related to the pressure of more rigorous education system. Then the incorrect posture of using eyes, reading and writing is also a risk factor for myopia. The third point is the lack of outdoor sports due to the growing popularity of electronic devices. Even after ten minutes of outdoor activities can stimulate the secretion of dopamine in the retina and prevent axial growth [32, 69, 82], so that it will induce the occurrence and progress of myopia.

In our study, we found another meaningful result. Children aged 6~11 years in both genders had the highest prevalence of astigmatism. Many studies reported that myopia might play a role in this acquired inattention, and in addition to congenital factors, is part of the natural cause [32, 34, 58, 69, 83]. The harm caused by astigmatism as following, such as blurred vision and head tilt for a clear binocular vision. Amblyopia is often caused by hyperopia astigmatism because it is not clear from far to near. Astigmatism was detected in 27.2 per 1000 male children with 6~11 age group in our study, it was much higher than other reports [28, 29, 45, 59, 71, 78, 84–87]. The higher incidence rate indicated that the serious causes of vision damage due to astigmatism needs more attention and relative earlier prevention for children. Since the measurement of astigmatism needs professional inspection, further epidemiolocal studies are necessary to obtain the data of astigmatism analysis in sub-regions.

Of the three estimates provided in this review, the prevalence of hyperopia had a decreasing trend in recent three decades. We believe that might be due to the lower number of studies in hyperopia analysis. However, the results of this meta-analysis propose the hypothesis that the decrease in the prevalence of hyperopia may be due to the increase in the prevalence of myopia in these years [88, 89]. As a cause of visual impairment and blindness, it should be the top priority as it has a profound impact on the productivity and quality of life of the individuals. Maintaining clear near vision is also important and can be easily corrected with reading glasses.

The meta-analysis of prevalence of URE in GBD sub-regions are interesting. The lowest and highest prevalence of URE was seen in South-East Asian and American children, respectively. The role of ethnic, genetic, and environ-mental factors should be taken into account [90]. Heterogeneity of the included studies was quite high, almost 100%, and due to this, low confidence is given to the pooled estimates. The reasons for these differences are not apparent. Heterogeneity can be due to differences in the methodology adopted or definitions used in the included studies. However, the quality assessment on the methodology adopted in the included studies were rated very high. Moreover, very close confidence intervals reported in the included studies suggest a low variance in the sample studied. It is also possible that prevalence of URE is inherently variable due to differences in socioeconomic status, urban or rural geographical location, and period of assessment. The prevalence and types of REs is subject to temporal trends [90, 91]. Considering the high quality of included studies, the pooled estimates were calculated for the three categories [92].

Lack of studies in many countries and lack of studies in each year in many countries were among the limitations of our study. Many studies were not included in the final analysis because they used different criteria for the detection of URE or because we only analyzed the studies published in English. An important limitation of many studies was that they did not use cycloplegic refraction in children which caused us limitations in the analysis of URE in individuals under 20 years of age. Although we tried to include studies with similar criteria in the analysis, these exclusion criteria may have biased the results. We did not evaluate different categories of refractive errors as low, moderate, or high myopia or hyperopia. Sparse data also limited the certainty of estimates of temporal trends and age patterns, particularly in children for URE. Despite the above limitations, this is the first study to show the overall prevalence of refractive errors according to WHO regions regardless of any categorization, which can be considered the most important advantage of the study. The development of refractive services, including the provision of affordable spectacles are the main strategies to address URE.

## Conclusions

Despite the above limitations, this is the first study to show the overall prevalence of refractive errors according to WHO regions regardless of any categorization, which can be considered the most important advantage of the study. The development of refractive services, including the provision of affordable spectacles are the main strategies to address URE.

## Supporting information

S1 Checklist (DOCX)

## Abbreviations

CI: confidence interval
GBD: Global Burden of Disease
PVA: presenting visual acuity
URE: Uncorrected refractive error
VI: visual impairment
